# Successful control of scleritis caused by *Nocardia farcinica*: A case report

**DOI:** 10.1097/MD.0000000000031481

**Published:** 2022-11-11

**Authors:** Zhongkai Hao, Hui Dang, Xin Gao, Chenming Zhang, Aijun Deng, Yue Tan, Gang Ding

**Affiliations:** a Department of Ophthalmology, School of Clinical Medicine, Weifang Medical University, Weifang, China; b Department of Ophthalmology, Jinan Second People’s Hospital, Jinan, China.

**Keywords:** eye infection, infectious scleritis, metagenomic detection, *N. farcinica*, pathogenic microorganism

## Abstract

*Nocardia farcinica* is an opportunistic pathogen that rarely causes ocular infections. This study reviewed a case of *N. farcinica*-mediated scleritis involving the corneal limbus.

**Patient concerns:**

A 56-year-old man was admitted to the Department of Keratology of Jinan Second People’s Hospital due to “a red and swollen right eye accompanied with severe pain for >1 month.” He denied any history of hypertension, diabetes, systemic immune diseases and eye surgery.

**Diagnoses:**

Corneal defect and scleral necrosis were observed by slit lamp. Combination of anterior segment optical coherence tomography and ophthalmic ultrasound biomicroscopy was used for diagnosis and evaluation of corneal and scleral conditions. Culture and metagenomic sequencing verified that the pathogen of scleritis was *N. farcinica*.

**Interventions:**

The patient was treated by sulfacetamide sodium eye drops, oral administration of sulfamethoxazole tablets, amikacin anterior chamber flushing, scleral debridement, and allogeneic scleral transplantation.

**Outcomes:**

The disease was successfully controlled.

**Lessons:**

Infectious scleritis caused by *N. farcinica* is extremely rare. Culture of pathogenic microorganisms remains to be the gold standard for the diagnosis of infectious eye diseases. Metagenomic sequencing shows potential promise in the diagnosis of infectious eye diseases. *N. farcinica* is sensitive to sulfonamides and amikacin.

## 1. Introduction

*Nocardia farcinica* is a bacterial pathogen causing diseases with clinically insidious onset, strong tissue damages and recurrence. It rarely causes severe purulent infection of the eyes,^[1]^ which can be manifested as keratitis,^[2]^ endophthalmitis,^[3]^ scleritis, and chorioretinitis.^[4]^ *N. farcinica* infection can be derived from exogenous sources, for example, trauma or endogenous sources, for example, hematogenous dissemination. The clinical symptoms lack specificity and immunocompromised population are susceptible to *N. farcinica* infection. Of the few reports of *N. farcinica* eye infection, the therapeutic efficacy of mild keratitis is acceptable, while the efficacy for endophthalmitis is relatively poor.^[3–5]^ In this study, we report a case of infectious scleritis involving the corneal limbus, which is suspected to be caused by *N. farcinica* in traumatic wound infection. The diagnosis was quickly and accurately confirmed by pathogenic microorganism culture and metagenomic sequencing. After treatment with frequent local use of sulfacetamide sodium eye drops, oral administration of sulfamethoxazole tablets, anterior chamber wash with amikacin, scleral debridement and allogeneic scleral transplantation, the disease was successfully controlled. To the best of our knowledge, this is the first reported case of infectious scleritis caused by *N. farcinica*.

## 2. Case report

**Figure 1.**
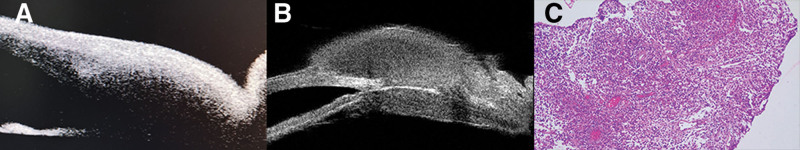
(a) OCT image of the anterior segment. Local bulge was observed in the nasal side of the bulbar conjunctiva near the limbus. The superficial corneal stroma was thinned. (b) UBM image. A mass was observed below the right nose near the limbus, and the internal echo was slightly low. The scleral echo was not continuous and was absent in some regions. (c) Pathological examination with HE stains. Acute and chronic inflammation with inflammatory granulation and tissue hyperplasia were observed in the lesions. OCT = optical coherence tomography, UBM = ultrasound biomicroscopy.

**Figure 2.**
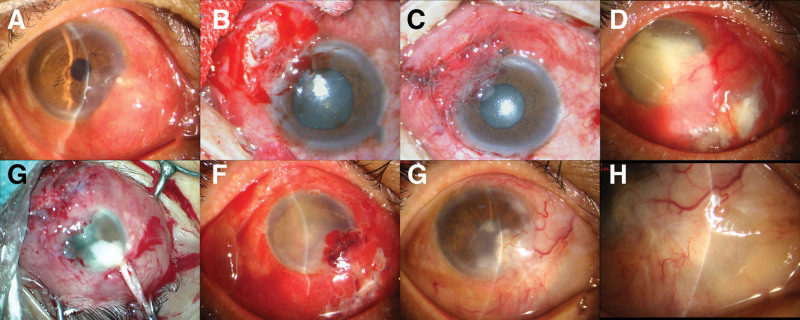
Photographs of the anterior segment in different period of treatment. (a) On admission, corneal infiltration and scleral bulging were observed (marks); (b) Chronic scleral necrosis and choroid were seen during the first operation; (c) Allogeneic scleral transplantation and conjunctival flap covering operation; (d) One month after the onset of the disease, there were multiple ulcers in the conjunctiva and a large amount of empyema in the anterior chamber; (e) A mass of pus in the anterior chamber was observed during the second operation (arrow); (f) Conjunctival hyperemia and corneal opacity were observed after second operation; (g and h) After 2 months of treatment, the conjunctival hyperemia was relieved, the cornea was clear, the anterior chamber was free of empyema, and the sclera was thinned.

The patient (male, 59 years old, decoration worker) had a red and swollen right eye accompanied with severe pain for >1 month. In the past, foreign bodies such as sediment were often collapsed into the eyes. The patient had no history of hypertension, diabetes, and systemic immune diseases. There was also no history of eye surgery. Eye examination showed that the left eye cornea had punctate residue of foreign bodies. Right eye conjunctiva had mixed hyperemia and the conjunctival sac had purulent secretions. There was a local bulge (with a size of 6 mm × 6 mm) in the sclera near the corneal limbus on the nasal side (Fig. 1b). There was a ring-shaped local infiltrate with clear borders and thinning of the matrix under the cornea (Fig. 1a). There was no empyema in the anterior chamber (Fig. 2a). Laboratory tests for rheumatoid factor were normal. X-ray examination showed no abnormality in both lungs. The concentration of γ-interferon was 101.85 pg/mL in the interferon-gamma release assay. The patient had weakly positive tuberculosis antibody. Tuberculin test was negative. During the time of waiting for the results of bacterial culture and drug susceptibility, the patients were treated with broad-spectrum antibiotics (levofloxacin hydrochloride eye drops 5 mL: 15 mg, tobramycin eye drops 0.4 mL: 1.20 mg) and immunosuppressive drugs (tacrolimus eye drops 5 mL: 5 mg), but the therapeutic effect was poor. Scleral exploration was then performed under local anesthesia. It was observed that scleral necrotic granulation reached the deep layer of the sclera. The choroid could be seen, and no obvious foreign body was observed (Fig. 2b). Allogeneic scleral lamellar transplantation and conjunctival flap covering operation were performed (Fig. 2c). The scleral lesion tissue was collected for pathological examination (Fig. 1c), bacterial culture and metagenomic sequencing. The results showed that bacterial culture was negative and sequencing results indicated the presence of *N. farcinica* (CGGTCATGTGCGGG GTCTGGTCCTGCAGCACCCGCAGCGTCGACA CCCAGTCCGGGCCGAACAGATCCGAGCGGG). *N. farcinica* was less concerned as a pathogen. Considering that bacteria culture was negative, and *N. farcinica* was a ubiquitous opportunistic pathogen and only immunocompromised population was susceptible, the patient was not diagnosed with pathogenic bacterial infection as patient’s immune function was normal. The patient’s condition improved after the operation.

**Figure 3.**
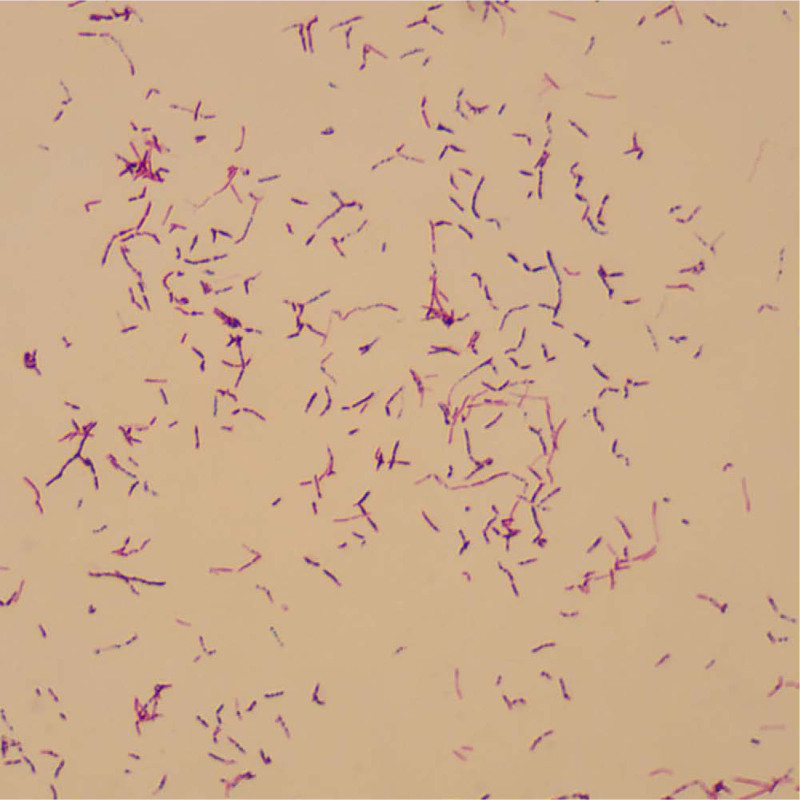
Mycelium is Gram-positive with a beaded appearance under light microscopy.

After 1 month of the treatment described above, the conjunctival mixed congestion was suddenly aggravated, and multiple subconjunctival abscesses, corneal edema, and anterior chamber empyema occurred around the transplanted sclera, and developed rapidly to cover the pupil area (Fig. 2d). Debridement of the conjunctiva and sclera, and anterior chamber flushing with amikacin (100 μg/0.1 mL) were immediately performed under local anesthesia. The purulent materials and liquid in the anterior chamber were collected for bacterial culture and metagenomic detection of pathogenic microorganism (Fig. 2e). Both bacterial culture and metagenomic sequencing results indicated the presence of *N. farcinica* (highly concerned) (Fig. 3). Thus, it was considered to be the infection with this pathogen. Based on the results of the drug susceptibility test, the patient was treated with sulfamethoxazole tablets (oral administration, 160 mg bid), sulfacetamide sodium eye drops (once/0.5 hour) and subconjunctival injection of amikacin (0.05 g, once a day for a total of 3 times). The condition was effectively controlled (Fig. 2f). Follow up (2 months) examinations showed that the patient’s condition was controlled. Conjunctival hyperemia was significantly reduced, the sclera was thinned, and the cornea had no obvious edema with good transparency and negative Tyndall effect. The hypopyon disappeared, the iris was normal, and the lens were covered with a small amount of white exuded membrane (Fig. 2g and h).

## 3. Discussion

*N. farcinica* is a Gram-positive, aerobic, filamentous actinomycete that is ubiquitous in soil, decaying vegetation, and water.^[6,7]^ It is the most commonly isolated species of *Nocardia*, accounting for 39.9%.^[8,9]^ *N. farcinica* is an opportunistic pathogen causing infection commonly in patients with immunodeficiency or long-term use of corticosteroids and immunosuppressants. People with normal immune function are also at risk of *N. farcinica* infection.^[10]^ Eye infection with *N. farcinica* is extremely rare. There are only 9 strains that are isolated from eyes among 441 different *Nocardia* strains collected by Wang et al.^[1]^ *N. farcinica* usually causes mild keratitis through exogenous routes (e.g., lens abrasion) or causes more severe endophthalmitis, invades the brain, lungs or other tissues, leading to purulent lesions through endogenous routes (e.g., hematogenous dissemination).^[11,12]^ Once the infection occurs, the onset of disease is acute, but the development is rapid with strong invasiveness, severe tissue destruction and long course of treatment.^[13]^ The patient in this study is a decoration worker. Because foreign bodies (e.g., soil) often collapsed into the eye, and the left cornea had punctate foreign body residues, it is suspected that the patient had infectious scleritis involving the corneal limbus caused by *N. farcinica* from the trauma wound.

Culture of pathogenic microorganisms is currently the gold standard for the diagnosis of infectious eye diseases. However, it takes a long time and has a low detection rate,^[14]^ which often affects the diagnosis and treatment of diseases. Therefore, we chose to combine culturing method with rapid and accurate metagenomic detection of tissue pathogenic microorganisms. Compared with traditional culturing method, it can be more rapid and precise to identify pathogenic bacteria and thus play a major role in the diagnosis of infectious eye diseases.^[15,16]^

Infectious scleritis caused by *N. farcinica* should be differentiated from the following diseases. First, scleral infection caused by *N. farcinica* lacks specific clinical manifestations. The fluffy exudate in the anterior chamber is usually similar to fungal infection and thus antifungal treatment is usually applied before diagnosis. In this study, fungi were not detected by fungal smear, confocal microscopy, microbial metagenomic sequencing and Gram staining, thus fungal infection was ruled out. Second, *N. farcinica* infection has similar clinical presentations (purulent scleritis with pain) to tuberculosis and the colony of *N. farcinica* is morphologically similar to that of mycobacteria.^[17]^ In this case, the tuberculosis antibody test was weakly positive, but the tuberculin test was negative. The lungs and systemic conditions were normal, thus tuberculous scleritis could be ruled out. Third, it must be differentiated from immune-related aseptic scleritis. This patient had no previous immune-related diseases, and examinations for systemic immune-related diseases were normal before and during this treatment. Thus, immune-related scleritis can be also excluded.

During eye exploration, scleral debridement was performed. In order to prevent eyeball rupture caused by scleral thinning, allogeneic scleral transplantation was performed to maintain the stability of the eyeball. In order to prevent corneal perforation due to the thinning of corneal stroma, additional conjunctival covering surgery was performed. However, it is unknown if aggravation after 1 month of stable disease condition was caused by the conjunctival covering surgery or the development of scleral infection. It is also unknown if the aggravation is due to the lack of attentions as a result of low concerns of pathogenic microorganisms in the metagenomic sequencing. According to previous studies and drug susceptibility results, *N. farcinica* is sensitive to compound sulfamethoxazole and amikacin, and resistant to cephalosporins, tobramycin and ciprofloxacin.^[5,18,19]^ It has been reported that vitreous body injection of amikacin may lead to macular necrosis.^[20,21]^ However, there are no effective antibiotics that can control infection through anterior chamber injection. In addition, the iris has significant inflammatory response. The iris septum associated with inflammatory exudates can prevent the drugs in anterior chamber from entering the vitreous body, thereby reducing the drug-induced macular toxicity.^[22]^ After the patient was treated with sulfacetamide sodium eye drops, oral administration of compound sulfamethoxazole tablets, and amikacin through anterior chamber flushing (100 μg/0.1 mL) and subconjunctival injection (once a day, 3 times in total), the condition of the disease was effectively controlled. After 2 months of treatment, the cornea became transparent, the number of endothelial cells was stable, and corneal damage was not serious. A small amount of white exudate membrane can be seen in front of the patient’s lens, which affects the vision. After the patient’s condition is stable, surgical intervention can be performed to improve the vision.

In this case report, we provided important understanding of infectious scleritis caused by *N. farcinica*. The disease caused by *N. farcinica* has insidious onset, severe tissue destruction, recurrence, and lacks specific clinical manifestations. It needs to be differentiated with fungal infection, tuberculosis, and immune-related aseptic scleritis. *N. farcinica* is sensitive to sulfonamides, quinolones, and amikacin. It takes a long time for the laboratory etiological test and the positive rate is low. Metagenomic sequencing should be considered to obtain fast and accurate diagnosis and provide timely treatment. However, it is still necessary to pay particular attention to the pathogenic microorganisms that are less concerned. Ocular disease caused by *N. farcinica* is extremely rare, and the case reported in this study enriched experiences and understanding on this disease. The case has been currently followed up for 2 months, and the condition is well controlled. Further observation is required to determine if it may recur. This case report also indicates that eye protection is very important during work, particularly those that may cause eye traumatic wound.

## Author contributions

ZH, CZ and AD were responsible for the conception and experimental design. ZH, HD and XG performed the experiments, analyzed and interpreted the data. ZH, CZ and AD were responsible for the writing and/or revision of the manuscript. YT and GD helped the analyzed and interpreted the data. CZ and AD provided the study supervision. All authors read and approved the final manuscript.

**Conceptualization:** Zhongkai Hao, Chenming Zhang, Aijun Deng.

**Data curation:** Zhongkai Hao, Hui Dang, Xin Gao.

**Formal analysis:** Zhongkai Hao, Hui Dang, Xin Gao, Chenming Zhang, Aijun Deng, Yue Tan, Gang Ding.

**Investigation:** Zhongkai Hao.

**Methodology:** Zhongkai Hao.

**Project administration:** Chenming Zhang, Aijun Deng.

**Resources:** Zhongkai Hao.

**Software:** Zhongkai Hao, Hui Dang.

**Supervision:** Yue Tan.

**Validation:** Zhongkai Hao, Hui Dang, Xin Gao, Chenming Zhang, Aijun Deng, Yue Tan, Gang Ding.

**Writing – original draft:** Zhongkai Hao.

**Writing – review & editing:** Chenming Zhang, Aijun Deng.
